# TDP-43 protein variants as biomarkers in amyotrophic lateral sclerosis

**DOI:** 10.1186/s12868-017-0334-7

**Published:** 2017-01-25

**Authors:** Stephanie M. Williams, Galam Khan, Brent T. Harris, John Ravits, Michael R. Sierks

**Affiliations:** 1 Chemical Engineering, The School for Engineering of Matter, Transport and Energy, Arizona State University, Tempe, AZ 85287-6106 USA; 2 Departments of Pathology and Neurology, Georgetown University Medical Center, Washington, DC 20057 USA; 3 Department of Neurosciences, University of California, San Diego School of Medicine, La Jolla, CA 92093-0624 USA

**Keywords:** Amyotrophic lateral sclerosis, TDP-43 variants, Plasma, Biomarker, Brain tissue, scFv

## Abstract

**Background:**

TDP-43 aggregates accumulate in individuals affected by amyotrophic lateral sclerosis (ALS) and other neurodegenerative diseases, representing potential diagnostic and therapeutic targets. Using an atomic force microscopy based biopanning protocol developed in our lab, we previously isolated 23 TDP-43 reactive antibody fragments with preference for human ALS brain tissue relative to frontotemporal dementia, a related neurodegeneration, and healthy samples from phage-displayed single chain antibody fragment (scFv) libraries. Here we further characterize the binding specificity of these different scFvs and identify which ones have promise for detecting ALS biomarkers in human brain tissue and plasma samples.

**Results:**

We developed a sensitive capture ELISA for detection of different disease related TDP-43 variants using the scFvs identified from the ALS biopanning. We show that a wide variety of disease selective TDP-43 variants are present in ALS as the scFvs show different reactivity profiles amongst the ALS cases. When assaying individual human brain tissue cases, three scFvs (ALS-TDP6, ALS-TDP10 and ALS-TDP14) reacted with all the ALS cases and 12 others reacted with the majority of the ALS cases, and none of the scFvs reacted with any control samples. When assaying individual human plasma samples, 9 different scFvs reacted with all the sporadic ALS samples and again none of them reacted with any control samples. These 9 different scFvs had different patterns of reactivity with plasma samples obtained from chromosome 9 open reading frame 72 (c9orf72) cases indicating that these familial ALS genetic variants may display different TDP-43 pathology than sporadic ALS cases.

**Conclusions:**

These results indicated that a range of disease specific TDP-43 variants are generated in ALS patients with different variants being generated in sporadic and familial cases. We show that a small panel of scFvs recognizing different TDP-43 variants can generate a neuropathological and plasma biomarker profile with potential to distinguish different TDP-43 pathologies.

**Electronic supplementary material:**

The online version of this article (doi:10.1186/s12868-017-0334-7) contains supplementary material, which is available to authorized users.

## Background

The progressive loss of motor neurons in regions including the spinal cord and cortex is a general occurrence in the advancement of the neurodegenerative disorder amyotrophic lateral sclerosis (ALS) [1–6]. Approximately 1–2 individuals per 100,000 are affected by ALS per year with an average life expectancy of 3–5 years [7, 8]. ALS cases can be divided into sporadic and familial classifications with the vast majority of the cases in the sporadic category [7, 8]. Mutations in genes including superoxide dismutase 1 (SOD1) and chromosome 9 open reading frame 72 (C9ORF72) have been linked to familial ALS [5, 7, 8]. Alterations to normal expression or structure of TAR DNA-binding protein 43 (TDP-43) have been suggested to play a major role in ALS, occurring in about 97% of cases [1, 3–6, 9, 10]. Normally TDP-43 is predominantly located in the cell nucleus where it participates as an important RNA/DNA binding protein involved in gene splicing and other RNA-related processes [5, 11]. However, during progression of ALS aggregates of TDP-43 accumulate in the cell cytoplasm. Variations of TDP-43 including ubiquitinated, truncated, phosphorylated and oligomeric forms exist in ALS [5, 12–14]. Therefore, reagents that can selectively bind different disease related TDP-43 variants have potential diagnostic and therapeutic applications for ALS.

An association between protein aggregation and disease development is also found in a number of other neurodegenerative disorders including Alzheimer’s (AD) and Parkinson’s (PD) diseases, where aggregates of beta-amyloid and alpha-synuclein, respectively, have been identified [15–31]. We previously demonstrated that we could generate single-chain variable fragments (scFvs) that selectively recognize different oligomeric variants of beta-amyloid, and that these scFvs could readily distinguish between human AD and control brain tissue, cerebrospinal fluid (CSF) and sera samples [28]. Similarly, we generated scFvs that selectively bind distinct oligomeric variants of alpha-synuclein, and demonstrated that these scFvs could readily distinguish between human PD and control brain tissue, CSF and sera samples [28]. These protein variant selective scFvs were isolated using an atomic force microscopy (AFM) based biopanning protocol [15, 20, 24–27]. The biopanning protocol utilizes a series of negative panning steps to remove phage particles that bind non-desired targets such as monomeric and fibrillar aggregates prior to completion of the positive panning step. This protocol was also utilized to generate scFvs against variants of TDP-43 present in human ALS cases (Stage 1A from Additional file 1: Fig. S1) [32]. We immunoprecipitated TDP-43 proteins from the homogenized motor cortex of ALS and control cases using a commercially available polyclonal anti-TDP-43 antibody as previously described [32]. Since TDP-43 pathology also exists in around 45% of frontotemporal dementia (FTD) cases [5, 33–37], we also included multiple rounds of negative panning against TDP-43 variants immunoprecipitated from the motor cortex of FTD cases. After biopanning, we identified 23 different complete scFv sequences that all preferentially bound ALS tissue over both FTD and healthy samples using indirect phage ELISAs (Stage 2 from Additional file 1: Fig. S1) [32].

Here we further characterized the 23 different scFvs to identify which ones reacted most strongly with ALS samples and to highlight the wide diversity of TDP-43 variants present in human neurodegenerative disease cases. We previously demonstrated a simple capture ELISA that utilizes a phage-displayed detection antibody with sub-femtomolar sensitivity in conjunction with capture scFvs similar to those examined here [28, 38]. Here we generated a phage based detection antibody against TDP-43 for use in a capture ELISA in conjunction with the 23 scFvs (Stages 1B and 2 from Additional file 1: Fig. S1) to analyze sporadic ALS, c9orf72 ALS, FTD and control human samples (Stage 3 from Additional file 1: Fig. S1).

## Results

### Selection of detection phage for capture ELISA

**Fig. 1:**
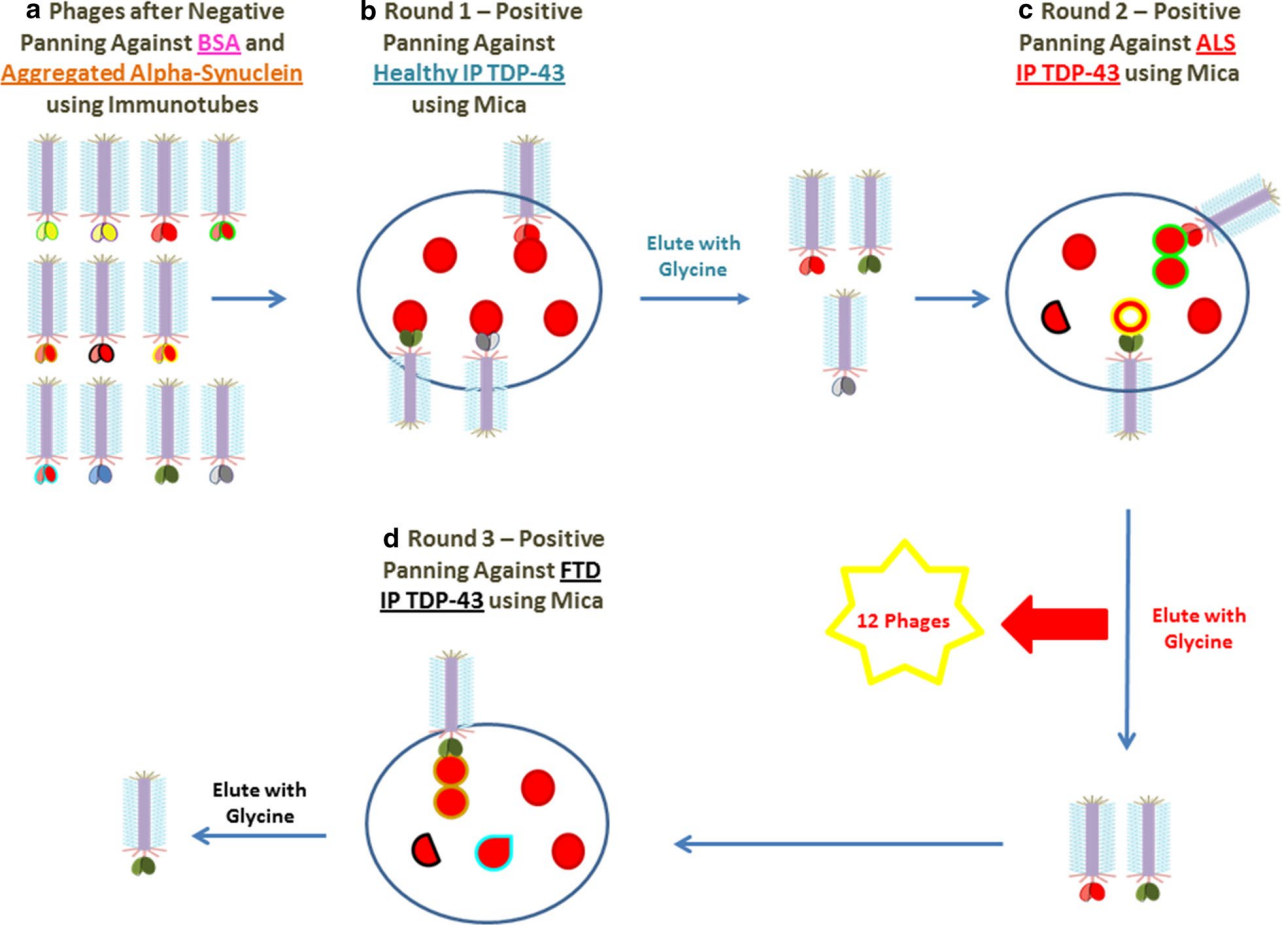
Schematic illustrating biopanning protocol to generate generic TDP-43 detection phages. To isolate detection phages that bind all healthy and disease related TDP-43 variants, we first eliminated all phage particles that bind non-desired targets including BSA and aggregated alpha-synuclein using immunotubes. Next, in the positive biopanning phase, the remaining phage particles were added to a mica surface containing TDP-43 immunoprecipitated from the motor cortex of healthy brain tissue. Any bound phages were glycine eluted and added to a mica surface with TDP-43 immunoprecipitated from ALS brain tissue. Following glycine elution the same manner of positive panning was repeated for TDP-43 immunoprecipitated from FTD brain tissue. Eluted phages were sequenced to verify scFv integrity and larger batches of each produced for further analysis

To obtain a detection scFv that binds to all TDP-43 variants including healthy and disease related species, we performed a series of AFM based negative and positive biopanning procedures. We first removed phages that bound off-target antigens including bovine serum album (BSA) and aggregated synthetic alpha-synuclein. We then performed sequential rounds of positive panning using TDP-43 immunoprecipitated from the motor cortex of healthy, ALS and FTD human brain tissue (Fig. 1). Following positive panning on a mica substrate containing TDP-43 immunoprecipitated from healthy brain tissue, bound phages were eluted and then added to mica coated with TDP-43 immunoprecipitated from ALS tissue. Following elution from the ALS tissue, the recovered phages were added to mica coated with TDP-43 immunoprecipitated from FTD tissue and eluted phage recovered. The recovered phage should bind healthy and disease variants of TDP-43 and were sequenced to verify the integrity of their DNA sequences.

**Fig. 2:**
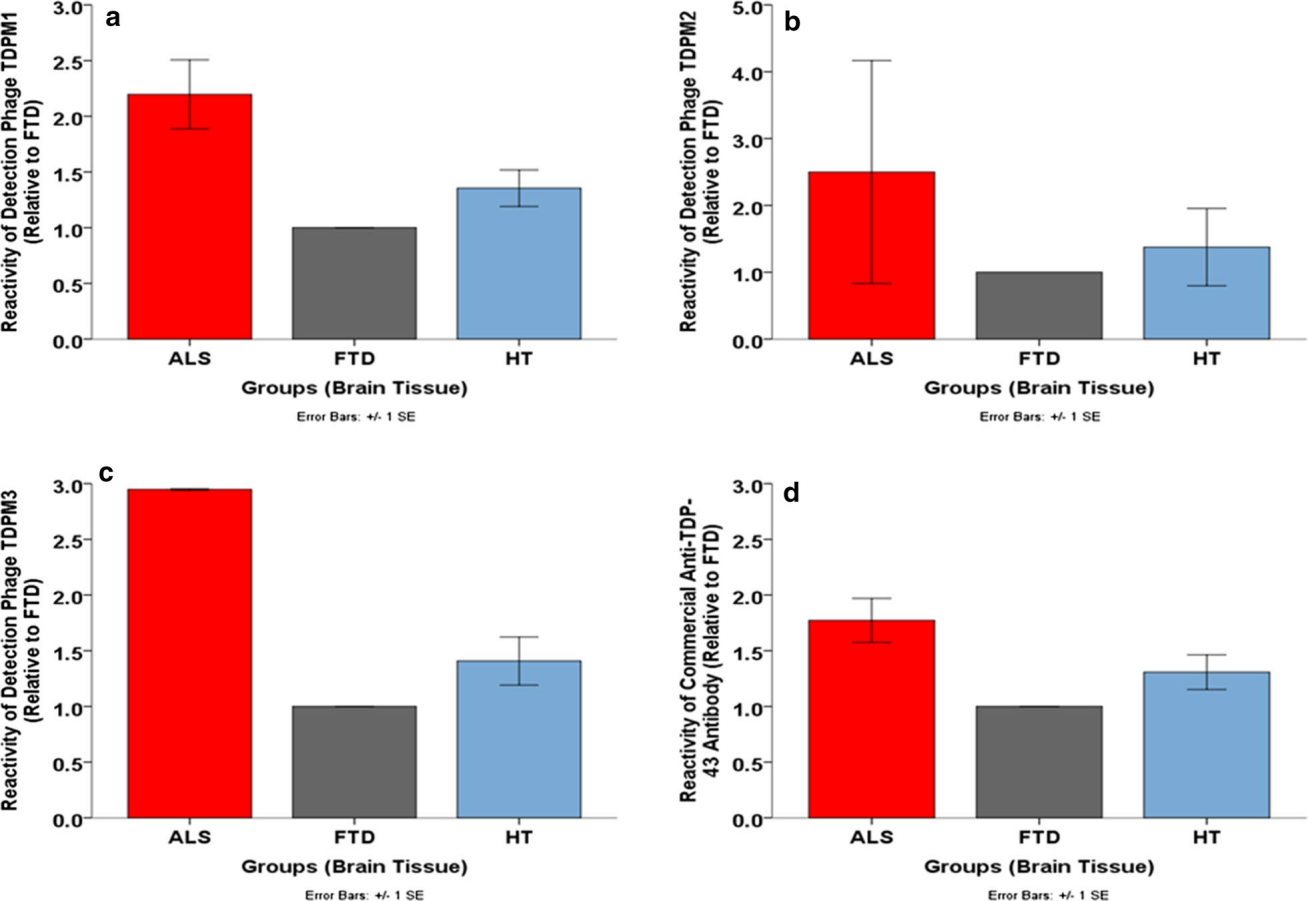
Selection of generic TDP-43 binding detection phages. Binding of selected phages with homogenized ALS, FTD and healthy control (HT) brain tissue from the motor cortex was tested in indirect phage ELISAs using three of the potential detection phages and a commercial anti-TDP-43 antibody. The TDP-43 binding patterns with **a** TDPM1, **b** TDPM2, **c** TDPM3 and **d** the commercial anti-TDP-43 antibody were all similar. TDPM1’s reactivity with all three groups seemed the closest to the commercial anti-TDP-43 antibody. Binding signals were represented relative to FTD. Bound phages were detected with an anti-M13-HRP antibody in these indirect ELISAs

**Fig. 3:**
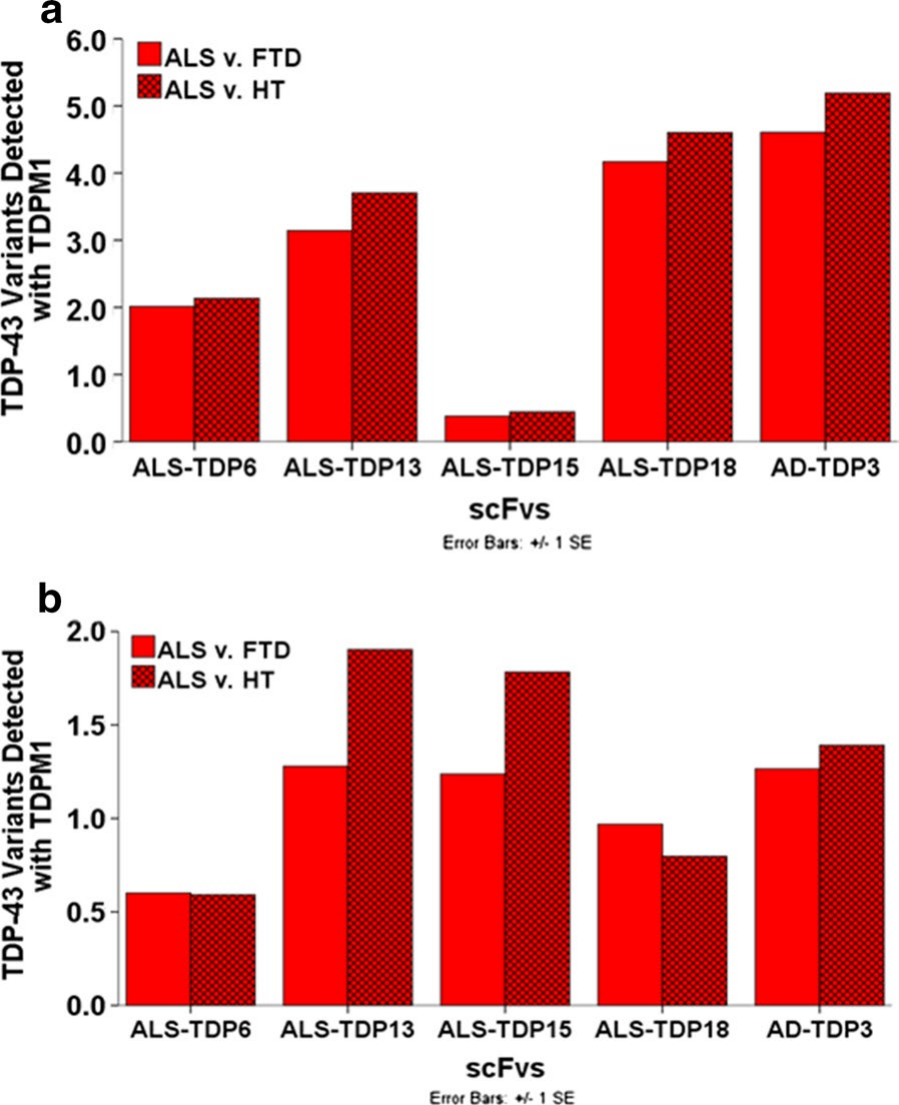
Validation of detection phage using capture ELISA with scFvs selective for ALS TDP-43 variants. Soluble antibody fragments from 5 (ALS-TDP6, ALS-TDP13, ALS-TDP15, ALS-TDP18 and AD-TDP3) of the 23 scFvs were produced and utilized as the capture scFvs in our phage capture ELISA. **a** TDP-43 immunoprecipitated from ALS, FTD and healthy brain tissue (motor cortex) was the antigenic source and bound antigens were detected using TDPM1. The results were represented as a difference between the ALS and both the FTD and control groups. TDPM1 produced strong signals with all 5 scFvs. **b** In a similar manner, substitution of brain tissue for the immunoprecipitated protein with all five capture scFvs and TDPM1 showed positive signals with ALS relative to both FTD and healthy tissue. Bound phages were detected with an anti-M13-HRP antibody in these capture ELISAs

We utilized indirect phage ELISAs to determine how well three of the different phages (TDPM1, TDPM2 and TDPM3) would bind to brain tissue samples from ALS, FTD and control cases (Fig. 2a–c) and compared the results with that obtained using a commercially acquired anti-TDP-43 antibody (Fig. 2d and Stage 2 from Additional file 1: Fig. S1). The TDPM1 phage most closely matched the binding of the control antibody in the phage ELISAs (Fig. 2a) so we selected this clone for use as the TDP-43 detection antibody in further analyses. We also verified that the TDPM1 phage worked as a detection antibody in a capture ELISA. We randomly selected five of the previously isolated scFvs against ALS TDP-43 variants (ALS-TDP6, ALS-TDP13, ALS-TDP15, ALS-TDP18 and AD-TDP3) for use as capture antibodies to assay TDP-43 immunoprecipitated from ALS, FTD and control tissue samples (Fig. 3a) and homogenized human brain tissue instead of immunoprecipitated proteins (Fig. 3b). Using TDPM1 as the detection phage, all five scFvs showed strong reactivity with the ALS samples compared to the FTD and control samples (Fig. 3a, b).

### Binding specificities of the anti-TDP-43 phages

Phage expressing the different anti-TDP-43 scFvs were previously selected for preferential binding to ALS brain tissue samples relative to both control and FTD cases as described [32]. We characterized the binding of 23 different phage-displayed scFvs toward ALS, FTD and healthy human brain tissue samples from the motor cortex using a simple indirect phage ELISA (Additional file 1: Fig. S1, Stage 2). The scFv clones were re-labeled in the current study to facilitate identification and simplify discussion. All scFvs were expressed in cell supernatant (Additional file 2: Fig. S2) and had stronger binding to ALS compared to both FTD and control samples (Additional file 3: Fig. S3) confirming the efficiency of our previously described biopanning protocol for generating reagents that bind disease specific protein variants [32].

### Recognition of multiple TDP-43 variants

**Fig. 4:**
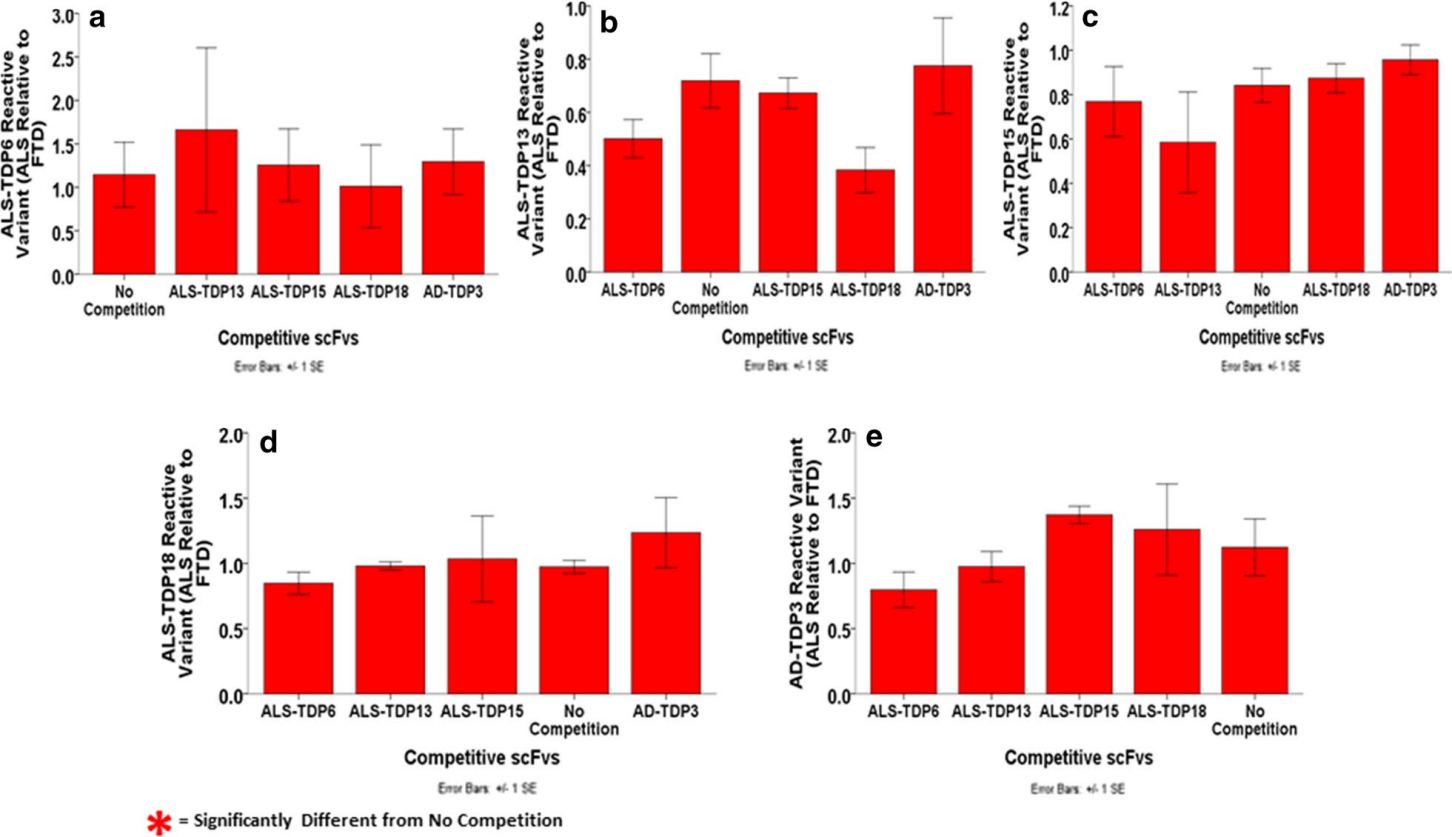
Competition ELISAs to test for overlapping epitopes recognized by the scFvs binding ALS selective TDP-43 variants. Competition capture ELISAs were utilized to determine if the scFvs bound similar epitopes. Samples of homogenized ALS, FTD and healthy brain tissue (motor cortex) were pre-incubated with the ALS-TDP6, ALS-TDP13, ALS-TDP15, ALS-TDP18 and AD-TDP3 scFvs individually for ~1 h. These samples were then utilized in capture ELISAs with the same five scFvs immobilized to the wells: **a** ALS-TDP6, **b** ALS-TDP13, **c** ALS-TDP15, **d** ALS-TDP18 and **e** AD-TDP3. Bound phages were detected with an anti-M13-HRP antibody and signal intensities calculated based on the difference between the ALS and FTD samples. All the scFvs produced similar binding signal strengths with or without the presence of the competitive scFvs. One-way ANOVA with LSD post hoc analysis supported the competitor scFvs’ inability to block the recognized antigenic site of our capture scFvs since no significant differences were detected between its presence and the no competition sample. Overall, these results demonstrated recognition of multiple TDP-43 variants by our scFvs

We next determined if the scFvs isolated from the ALS biopanning process were binding distinct or similar or overlapping epitopes. We performed competition ELISAs utilizing the same five scFvs randomly selected previously (ALS-TDP6, ALS-TDP13, ALS-TDP15, ALS-TDP18 and AD-TDP3). Addition of competing scFvs did not significantly reduce the detection signal indicating that the five scFvs all bind separate epitopes (Fig. 4a–e). While we did not perform competition ELISAs with all 23 scFvs, based on the competition ELISA results obtained with these five scFvs (Fig. 4a–e) and the different binding intensities of the 23 phages (Additional file 3: Fig. S3), it is likely that an array of different ALS associated TDP-43 epitopes are recognized by the different scFvs.

### Selection of individual ALS cases utilizing human brain tissue

**Fig. 5:**
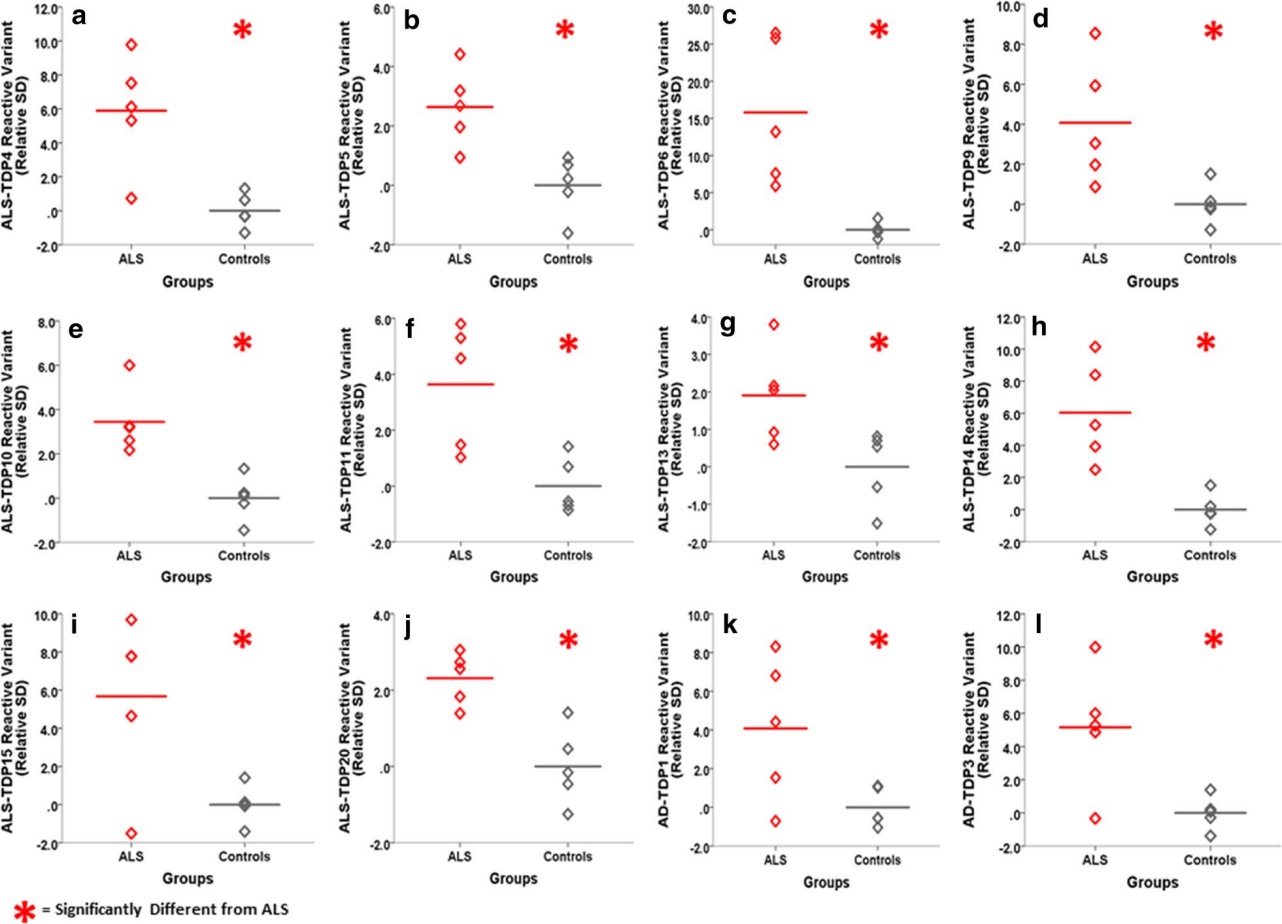
Characterization of individual ALS brain tissue samples with the different scFvs. Assessment of the binding specificities of the scFvs with brain tissue (motor cortex) from 5 individual ALS cases and 5 controls (as opposed to with pooled samples used in past studies). Results with the 12 most reactive scFvs are shown: **a** ALS-TDP4, **b** ALS-TDP5, **c** ALS-TDP6, **d** ALS-TDP9, **e** ALS-TDP10, **f** ALS-TDP11, **g** ALS-TDP13, **h** ALS-TDP14, **i** ALS-TDP15, **j** ALS-TDP20, **k** AD-TDP1 and **l** AD-TDP3. Here we utilized a biotinylated TDP-43 phage capture ELISA system to enhance our detection capabilities. On average, all 12 scFvs showed significantly greater reactivity with the 5 ALS brain tissue samples compared to the controls. Statistical significance is based on one-way ANOVA with LSD post hoc analysis at p < 0.05. Signal intensities are based on the number of standard deviations each sample is from the controls. This is calculated by subtracting the mean of the controls from each sample and then dividing the result by the SD of the controls

While the previous studies utilized pooled aliquots to conserve limited sample availability, selection of scFvs that bind all or different sets of ALS cases would be beneficial for biomarker analysis. To study individual ALS cases we first purified each of the scFvs using Ni–NTA columns (Additional file 4: Fig. S4) and then analyzed 5 ALS and 5 control homogenized brain samples individually. Results from 12 scFvs yielding the strongest reactivity with the individual samples are shown (Fig. 5a–l) where the results are represented as number of standard deviations relative to the controls. As expected, all 12 scFvs had significantly greater binding preference for ALS relative to the controls (Fig. 5a–l). The reactivity of each sample with each scFv is shown (Additional file 5: Table S1) where each plus sign indicates an additional one standard deviation increase from the mean of the control (only sample with more than 2SD were highlighted). Three scFvs (ALS-TDP6, ALS-TDP10 and ALS-TDP14) reacted with all 5 ALS samples, five (ALS-TDP4, ALS-TDP5, ALS-TDP9, ALS-TDP15 and AD-TDP3) reacted with 4 of the 5 ALS samples and four (ALS-TDP11, ALS-TDP13, ALS-TDP20 and AD-TDP1) reacted with 3 of the 5 ALS cases. While each scFv showed different binding specificity to the different ALS samples, none of the scFvs reacted with any of the control samples. Because the binding profiles of the scFvs with the different ALS cases varied, this provides further support that the different scFvs bind distinct TDP-43 antigens.

### Selection of individual ALS cases utilizing human plasma

**Fig. 6:**
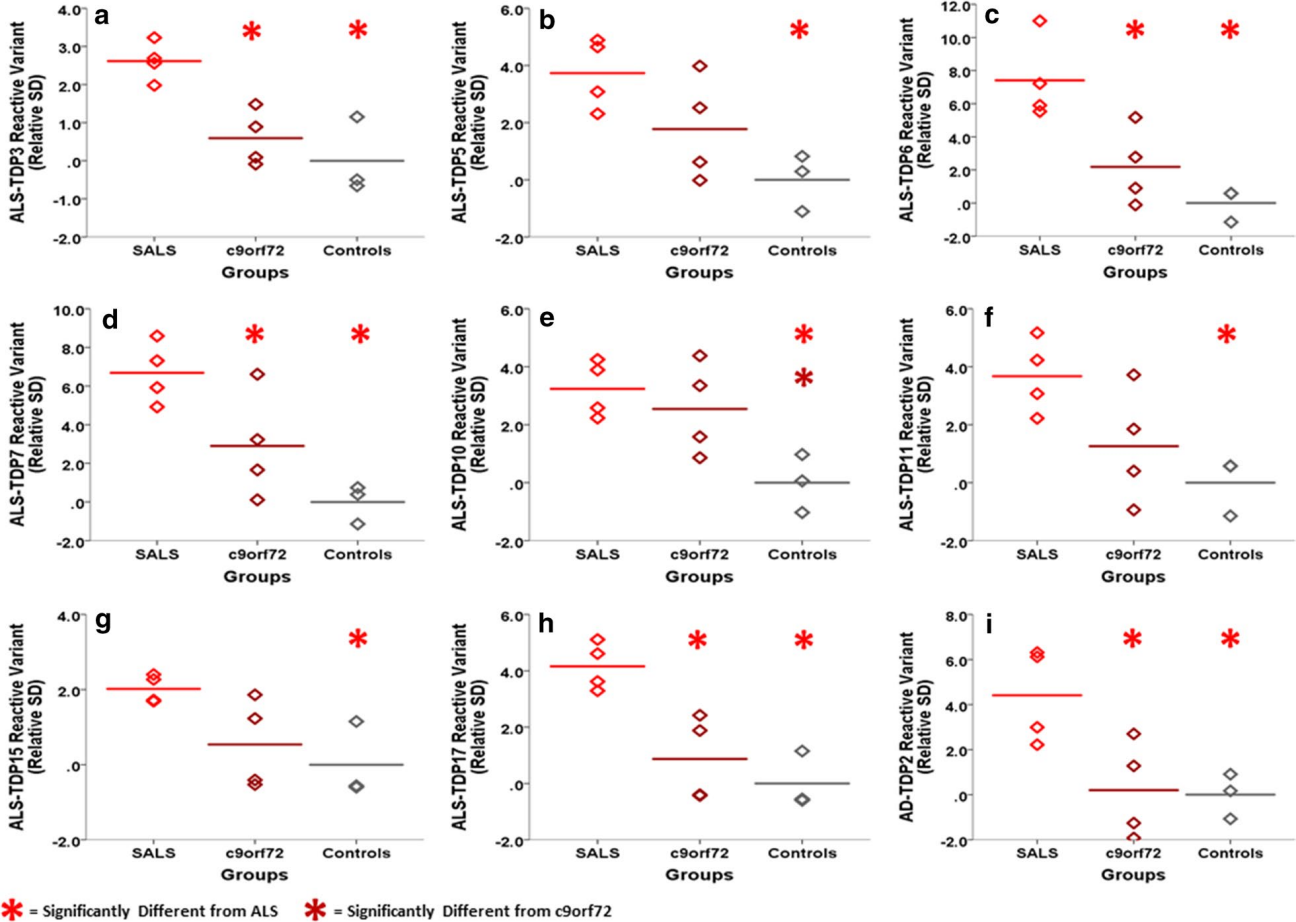
Characterization of individual ALS plasma samples with the different scFvs. Human plasma samples from 4 sporadic ALS cases, 4 c9orf72 ALS cases and 3 healthy control cases were assayed for the presence of TDP-43 variants. Results of the 9 most reactive scFvs are shown: **a** ALS-TDP3, **b** ALS-TDP5, **c** ALS-TDP6, **d** ALS-TDP7, **e** ALS-TDP10, **f** ALS-TDP11, **g** ALS-TDP15, **h** ALS-TDP17 and **i** AD-TDP2. A biotinylated TDP-43 phage capture ELISA system was utilized to enhance our detection capabilities. On average, all the scFvs showed significantly greater reactivity with the sporadic ALS cases relative to the controls. ScFvs ALS-TDP3, ALS-TDP6, ALS-TDP7, ALS-TDP17 and AD-TDP2 also showed statistical differences between the sporadic ALS cases and the c9orf72 ALS samples. In addition, ALS-TDP10 could significantly distinguish between the c9orf72 ALS and control groups. Statistical significance is based on one-way ANOVA with LSD post hoc analysis at p < 0.05. Signal intensities are based on the number of standard deviations each sample is from the controls. This is calculated by subtracting the mean of the controls from each sample and then dividing the result by the SD of the controls

Since the scFvs isolated against TDP-43 variants selectively present in human ALS but not healthy brain tissue could distinguish between ALS and control brain tissue samples, we next determined if any of the scFvs could also distinguish amongst sporadic ALS, c9orf72 ALS and control plasma samples. Here we tested individual plasma samples from 4 sporadic ALS cases, 4 ALS c9orf72 cases and 3 controls. Results from the nine scFvs that produced significant differences between the sporadic ALS and control plasma cases are shown (Fig. 6a–i). Five of the nine scFvs (ALS-TDP3, ALS-TDP6, ALS-TDP7, ALS-TDP17 and ALS-TDP2) also produced statistical differences between the sporadic ALS samples and the c9orf72 ALS cases. While most of the scFvs showed lower binding with the c9orf72 ALS samples compared to the sporadic ALS samples, the ALS-TDP10 scFv was the only scFv that also showed significantly higher reactivity with the c9orf72 ALS samples compared to the control group (Fig. 6e). The strength of the signals obtained with each of the scFvs toward each sample is shown where the first “+” indicates more than 1.5 SD increase from mean of the controls and each additional “+” sign indicating an additional 1 SD increase (Additional file 6: Table S2). While all 9 scFvs reacted with each of the 4 sporadic ALS plasma samples but none of the controls, the binding profile with the c9orf72 ALS cases varied between scFvs. For example, two of the scFvs (ALS-TDP7 and ALS-TDP10) reacted with 3 of the 4 c9orf72 ALS cases, 4 of the scFvs (ALS-TDP5, ALS-TDP6, ALS-TDP11, ALS-TDP17) reacted with 2 of the 4 c9orf72 ALS cases, while 2 (ALS-TDP15 and AD-TDP2) reacted with 1 of the 4 c9orf72 ALS cases, although not the same one. ALS-TDP3 did not produce strong reactivity with any of the c9orf72 ALS cases. This variation in binding intensities and sample selection again supports a difference in target recognition between the scFvs. Additionally, ALS-TDP5, ALS-TDP6, ALS-TDP10, ALS-TDP11 and ALS-TDP15 produced the best results with both the individually tested ALS brain tissue and plasma cases (Additional file 5: Table S1, Additional file 6: Table S2) indicating their potential value as biomarkers for ALS.

## Discussion

While TDP-43 pathology is present in the vast majority of examined ALS cases [1, 3–6, 9, 10, 39], the conformation and location of the TDP-43 aggregates can vary, including oligomeric, truncated, phosphorylated and ubiquitinated configurations [12, 13]. Isolation of reagents that can selectively recognize disease relevant TDP-43 variants could facilitate diagnosis of ALS, particularly if these variants could be detected in blood based samples during early even pre-symptomatic stages of disease progression [40–42]. We previously isolated scFvs with preferential reactivity for TDP-43 immunoprecipitated from ALS human brain tissue relative to TDP-43 immunoprecipitated from healthy and FTD human brain tissue using our AFM based biopanning procedures [32]. Here we further identify a subset of these scFvs with diverse binding specificities that have potential applications to detect blood-based biomarkers for ALS.

We previously identified 23 distinct valid scFv sequences from the ALS TDP-43 positive biopanning and all showed preferential reactivity for homogenized human ALS brain tissue samples compared to FTD and control cases (Additional file 3: Fig. S3). We also generated a phage-displayed scFv that binds all forms of TDP-43 including ALS, FTD and healthy variants (Fig. 2a) to be utilized in our capture ELISA protocol. We applied competition ELISAs to show that the different scFvs generated against the ALS relevant TDP-43 variants were binding different epitopes (Fig. 4a–e). Binding intensity variations between all 23 phages in the indirect phage ELISAs (Additional file 3: Fig. S3) also suggest the presence of multiple TDP-43 variants in ALS.

Using the scFvs in a capture ELISA format, we showed that 12 of the scFvs can statistically distinguish between ALS and healthy human brain tissue samples (Fig. 5a–l). By plotting signal strength as a function of number of standard deviations from the control, we showed that three of the scFvs (ALS-TDP6, ALS-TDP10 and ALS-TDP14) reacted with all five ALS samples studied but none of the controls, while the nine other scFvs selected different combinations of 3–4 of the ALS cases and again none of the controls (Additional file 5: Table S1). The variation in signal intensities for each sample suggests that the different targeted TDP-43 variants are present at different concentrations in the ALS cases. We have previously illustrated successful identification of AD and PD sera cases using multiple scFvs [28] and expect that such an approach can also be used for diagnosis of ALS.

To determine their potential diagnostic value, we analyzed plasma samples from 4 sporadic ALS cases, 4 c9orf72 ALS cases and 3 controls using the anti-TDP-43 scFvs and identified 9 promising scFvs. All 9 of the scFvs reacted with all 4 sporadic ALS cases (Additional file 6: Table S2). ALS-TDP7 and ALS-TDP10 selected 3 of the 4 c9orf72 ALS cases, 6 of the 9 scFvs recognized 1–2 of the c9orf72 ALS cases and ALS-TDP3 did not select any of the c9orf72 ALS cases. Since the panning protocols included exhaustive negative panning against healthy control human samples, as expected, none of the scFvs reacted with any of the control samples. Since more than 23 scFvs were generated from the TDP-43 panning process, it is possible that screening some of the additional remaining scFvs will identify antibody fragments that select all of the c9orf72 ALS cases. Since plasma samples from different patients show different reactive profiles with the panel of anti-TDP scFvs, each patient may likely have their own personal biomarker profile corresponding to subtly different types of ALS. Utilizing a combination of multiple anti-TDP-43 scFvs may prove valuable in providing a personalized diagnosis for each patient in both sporadic and familial ALS cases. A personalized blood-based diagnosis system could also be very helpful for initiating and monitoring treatment strategies for ALS [41, 42].

Here we characterize the binding specificities of a subset of 23 anti-TDP-43 scFvs we previously generated [32] with both human brain tissue and plasma samples to illustrate their preferential reactivity with ALS. The scFvs generated by selectively biopanning for TDP-43 variants present in ALS but not FTD brain tissue do preferentially bind ALS compared to both FTD and control samples. These results further support the proficiency of our biopanning process at isolating reagents reactive with target variants of interest. In future studies we intend to screen plasma from larger ALS and FTD sample sets to reaffirm which scFvs are ALS specific and if any also cross-react with FTD samples. Also, since TDP-43 pathology is not unique to ALS and FTD, but has been detected in other neurodegenerative disease including ~ 57% of individuals with AD, 19% of PD cases, 45% of individuals having dementia with Lewy bodies (DLB) and 100% of tested Huntington’s cases [12, 13, 39, 43–47], we also intend to screen the scFvs for cross reactivity with these other diseases. If scFvs can selectively bind specific disease cases, they may be useful reagents along with reagents to other protein variants including beta-amyloid, alpha-synuclein and tau to help improve diagnosis of different neurodegenerative diseases [48].

We have previously shown that scFvs against alpha-synuclein variants associated with Parkinson’s disease have excellent therapeutic value [49, 50]. In a similar fashion, since our scFvs selectively bind ALS associated variants of TDP-43, in addition to diagnostic value, the scFvs may also have potential therapeutic value.

## Conclusion

Our TDP-43 reactive scFvs were selective for individual ALS cases relative to controls utilizing both human brain tissue and plasma samples. The scFvs recognized both sporadic and familial ALS cases with plasma. These results support the potential diagnostic value of our TDP-43 scFvs when employed in our phage capture ELISA system.

## Methods

### Human samples

ALS, FTD and healthy human brain tissue samples from the motor cortex and immunoprecipitated TDP-43 proteins from these cases were provided from the Georgetown Brain Bank (Georgetown University Medical Center) and New York Brain Bank (Columbia University). Patient age and gender for these samples are provided in Table 1. All samples were provided de-identified with regard to patient identifiable information, and all tissue/biofluid banks are operating under institutional IRB guidelines. Plasma samples from ALS, c9orf72 ALS and healthy human cases were provided by Dr. John Ravits (University of California, San Diego School of Medicine) and collected by an Investigational Review Board-compliant process. Patient age and gender for these samples are provided in Table 1.

**Table 1:** Patient cohort demographics

Cases	Age	Gender	Sample type
ALS-1	88	F	BT
ALS-2	60	M	BT
ALS-3	73	M	BT
ALS-4	45	M	BT
ALS-5	64	M	BT
FTD-1	80	M	BT
FTD-2	66	M	BT
FTD-3	67	F	BT
FTD-4	74	M	BT
FTD-5	68	M	BT
C-1	79	M	BT
C-2	90	M	BT
C-3	41	M	BT
C-4	60	M	BT
C-5	72	M	BT
SALS-1	58	M	P
SALS-2	38	M	P
SALS-3	58	F	P
SALS-4	46	M	P
c9orf72-1	73	M	P
c9orf72-2	61	M	P
c9orf72-3	58	F	P
c9orf72-4	67	F	P
C-1	62	M	P
C-2	50	M	P
C-3	41	F	P

*BT* brain tissue, *P* plasma

### Phage production

To produce phage particles from the ALS TDP-43 clones isolated earlier, we essentially follow the previously described protocol (http://www.lifesciences.sourcebioscience.com/media/143421/tomlinsonij.pdf) [28, 32, 38]. *E. coli* TG1 cells containing the plasmids for our clones were cultured in 2xYT containing 100 μg/ml ampicillin and 1% glucose until OD_600_ was 0.4–0.6. The cells were then incubated with 2 × 10^11^ of KM13 helper phage or hyperphage (Progen, Germany) for 30 min without shaking, followed by media exchange to 2xYT containing 100 μg/ml ampicillin, 50 μg/ml kanamycin and 0.1% glucose post centrifugation. The cells were then cultured overnight at 30 °C, followed by centrifugation to isolate the supernatant. Polyethylene glycol (PEG)/NaCl was added to the supernatant and it incubated on ice for 1 h. The mixture was then centrifuged and the pellet resuspended in PBS. Following another 1 h incubation on ice, additional cell debris was removed via a last centrifugation step. Their concentrations were estimated using a bicinchoninic acid (BCA) assay (Pierce, USA) and stored at −80 °C.

### Biopanning for anti-TDP-43 detection antibody

For the capture ELISA utilized here we require a detection scFv that recognizes all forms of the target antigen, in this case TDP-43. The detection scFv is displayed on the phage surface generating essentially a self-assembling nanoparticle for detection. The detection antibody should bind multiple forms, conformations and variants of the target TDP-43 antigen. To acquire the detection antibody, we utilized our previously described AFM based biopanning protocols [32]. We utilized a combination of three different scFv libraries including the Tomlinson I and J libraries and Sheets library [51] as our initial scFv pool. A series of negative panning steps were then completed to remove phage binding non-desired targets including bovine serum albumin (BSA) and aggregated synthetic alpha-synuclein (Fig. 1). We then performed a positive panning step using an aliquot of TDP-43 immunoprecipitated from the motor cortex of healthy human brain tissue deposited on a mica substrate (Fig. 1). Bound phages were eluted with glycine and added to a second piece of mica containing an aliquot of TDP-43 immunoprecipitated from ALS human brain tissue. Following glycine elution, phages were then added to a third piece of mica containing an aliquot of TDP-43 immunoprecipitated from FTD human brain tissue. Bound phages were again eluted with glycine and recovered by infection of TG1 cells. We utilized multiple rounds of positive panning with TDP-43 immunoprecipitated from diverse brain homogenate samples to ensure selection of a detection antibody that is reactive with normal and disease associated forms of TDP-43. Eluted phages were then screened using phage ELISAs and the integrity of their DNA sequences verified (Stage 2 from Additional file 1: Fig. S1). Plasmid isolation was accomplished using the Qiagen Miniprep Kit (Valencia, CA, USA). The selected TDP-43 detection phage was then biotinylated using the EZ-Link Pentylamine-Biotinylation kit (Thermo Scientific, USA) as previously described [38] for use in the capture ELISA.

### Indirect ELISAs

Indirect ELISA and tissue homogenization were performed as described previously [28, 32, 38]. Briefly, 2–10 μg/ml of homogenized human brain tissue was added to a 96-well ELISA plate and incubated for 1 h at 37 °C. Following three washes with 0.1% PBS-Tween 20, non-specific binding sites were blocked with 2% milk in PBS. Either a 1/100 dilution of phage particles or 1/1000 of rabbit anti-TDP-43 antibody (ProteinTech, IL, USA) was added to the wells followed by anti-M13 HRP (GE Healthcare Life Science, NJ, USA) or goat anti-rabbit IgG HRP (Santa Cruz Biotechnology, Texas, USA), respectively. Enzyme detection was achieved using the SuperSignal ELISA Femto Maximum Sensitivity Substrate kit (Thermo Scientific, USA) and signal intensities quantified using the Perkin Elmer Wallac 1420 Victor2 Multilabel Counter.

### ScFv production and purification

ScFvs were expressed and purified as previously described [28, 32, 38]. Briefly, HB2151 cells were cultured for 2–3 h at 37 °C in 2xYT, 0.1% glucose and ampicillin until OD_600_ was 0.4. Isopropyl β-d-1-thiogalactopyranoside (IPTG) was then added and the temperature reduced to 30 °C. The following day the supernatant was harvested and stored at −20 °C. Purification was completed under native conditions by incubating the supernatant with Ni–NTA agarose beads (Qiagen, CA, USA) for 1–2 h at 4 °C. The mixture was then transferred to a column. Following washing, bound scFvs were eluted using a 250 mM imidazole solution. Dot and Western blotting analyses were used to confirm expression and purification using the C-Terminal c-myc tag on our scFvs.

### Phage capture ELISAs

Phage capture ELISAs were performed essentially as described previously [38]. Briefly, unconcentrated supernatant containing the scFvs or Ni–NTA purified scFvs was added to the wells of high binding ELISA plates for 1 h at 37 °C on a shaker. All incubations were performed at 37 °C whilst shaking. After washing with 0.1% PBST three times, the wells were blocked with 2% milk in PBS. Next, human brain tissue (2–10 μg/ml) or sera (1/100 v/v) was added followed by 200 ng/ml of the 40 mmol carboxyl biotinylated phage. After addition of the secondary antibody avidin-HRP (Sigma-Aldrich, USA), enzyme detection was again achieved using the SuperSignal ELISA Femto Maximum Sensitivity Substrate kit.

When screening the potential detection phage using the capture ELISA system, the phage was not biotinylated and therefore detected using the anti-M13 HRP antibody. Similarly, any reactivity with the commercial anti-TDP-43 antibody was recognized with the goat anti-rabbit IgG HRP secondary. Lastly, the quantity of immunoprecipitated proteins utilized in these ELISAs was 20 ng.

### Competition ELISAs

Competition ELISAs were carried out using the phage capture ELISA protocol except preceding addition of the brain tissue, the samples were pre-incubated with the different competitive scFvs for ~1 h.

### Statistical analysis

The ratio of each sample reading to the value obtained for PBS was first calculated. The mean of the control and/or FTD cases was then subtracted from each sample. This type of calculation was utilized due to the difficulty in acquiring large purified quantities of the targets recognized by our scFvs in order to generate a standard curve to quantify the reactivity of each sample. We evaluated the reactivity of the test sample relative to the average signal intensity of the control and/or FTD groups by subtracting their average reactivity from that of the ALS group thus setting control values to zero. Statistical significance was calculated using one-way ANOVA and LSD Post-Hoc analyses with significance at p < 0.05. The graphs and statistical analyses were completed using the IBM SPSS Statistics 23 program. In Figs. 5 and 6, to illustrate the intensity difference between the test and control groups, we plotted the number of standard deviations each sample was from the controls by subtracting the mean of the controls from each sample and dividing the result by the standard deviation of the controls. In Additional file 5: Table S1, Additional file 6: Table S2, the level of activity was further highlighted using a “+” sign system. In Additional file 5: Table S1 each “+” sign indicated one standard deviation (SD) increase relative to the mean of the controls. In Additional file 6: Table S2 the first “+” indicated a 1.5 SD increase followed by each additional “+” sign indicating another 1 SD increase. Furthermore, since brain tissue homogenization was conducted at different intervals, technique variation was accounted for in the ELISAs by first dividing by matching controls.

## Additional files

**Additional file 1: Figure S1.** Schematic of the Development of our Biotinylated TDP-43 Phage Capture ELISA System. A schematic of the entire process utilized to develop our biotinylated TDP-43 phage-capture ELISA system for heightened detection of TDP-43 variants is shown. Stage 1A—Previously described AFM based negative and positive biopanning protocol to isolate the capture scFvs reactive with TDP-43 variants isolated from ALS brain tissue [32]. Starting with an initial scFv library, we first eliminated scFvs reactive with undesired targets including BSA, aggregated alpha-synuclein and healthy human brain tissue using immunotubes. AFM analysis was utilized to monitor the process. We next completed negative panning against TDP-43 immunoprecipitated from healthy and FTD brain tissue (motor cortex) using a mica surface to conserve limited sample availability. The remaining phages were then utilized in positive biopanning against TDP-43 immunoprecipitated from ALS brain tissue. Any eluted phages following this process should be specific for ALS related TDP-43 variants. Stage 1B—To isolate a detection phage that is reactive with all forms of TDP-43 we utilized the AFM biopanning process described in Fig. 1. Stage 2—Both the potential ALS TDP-43 reactive phages isolated in the previous study and the detection phages acquired in the current study were first screened in indirect ELISAs against ALS, FTD and healthy brain tissue. Any bound phage particles were detected with an anti-M13-HRP secondary antibody. ALS TDP-43 specific phages should generate signals only in the wells containing ALS tissue, whilst the detection phages should produce signals with all three tissue types. Stage 3 - Finally, following biotinylation of the 2700 coat protein on our detection phage to heighten our sensitivity, our complete phage capture ELISA system was used to analyze individual human brain tissue and plasma samples. Some of the utilized illustrations were adapted from previously published graphics [32, 38].

**Additional file 2: Figure S2.** Expression of Soluble Antibody Fragments. Soluble antibody fragments from 17 of the 23 scFvs were produced and secreted into the supernatant as confirmed by dot blot analysis using an anti-c-myc antibody to label the scFvs. A scFv reactive with phosphorylase B was included as a positive control in the assay.

**Additional file 3: Figure S3.** Signal Intensities of 23 Different Phages with Preferential Reactivity for ALS Related TDP-43 Variants. Indirect phage ELISAs using 23 different previously isolated clones with preferential reactivity for ALS TDP-43 variants were completed with homogenized brain tissue from ALS, FTD and healthy samples (motor cortex). All the phages generated increased reactivity with ALS brain tissue compared to both FTD and control cases. The ratio of ALS to control tissue for each phage was first calculated, followed by subtraction of the binding ratio produced with FTD brain tissue. The results of ALS relative to FTD are shown. Bound phages are detected with an anti-M13-HRP antibody.

**Additional file 4: Figure S4.** Analysis of Purified Soluble Antibody Fragments by Western Blot. Following Ni–NTA purification of 17 of the 23 scFvs, analysis by Western blotting indicated the presence of the expected ~28 kDa band corresponding to a full length scFv (except for ALS-TDP10). The estimated molecular weight for each scFv is indicated below their respective band.

**Additional file 5: Table S1.** TDP-43 Protein Variants in Human Brain Tissue.

**Additional file 6: Table S2.** TDP-43 Protein Variants in Human Plasma.

## Abbreviations

ALS: amyotrophic lateral sclerosis
AFM: atomic force microscopy
FTD: frontotemporal dementia
scFv: single chain antibody fragment
SOD1: superoxide dismutase 1
c9orf72: chromosome 9 open reading frame 72
TDP-43: TAR DNA-binding protein 43
AD: Alzheimer’s disease
PD: Parkinson’s disease
CSF: cerebrospinal fluid
BSA: bovine serum album
DLB: dementia with Lewy bodies
PEG: polyethylene glycol
BCA: bicinchoninic acid
IPTG: isopropyl β-d-1-thiogalactopyranoside
SD: standard deviation
