# Supplementary figures and images for: TDP-43 protein variants as biomarkers in amyotrophic lateral sclerosis
Source: BMC Neurosci. 2017 Jan 25;18:20. doi: 10.1186/s12868-017-0334-7 (PMC5264476; doi:10.1186/s12868-017-0334-7)

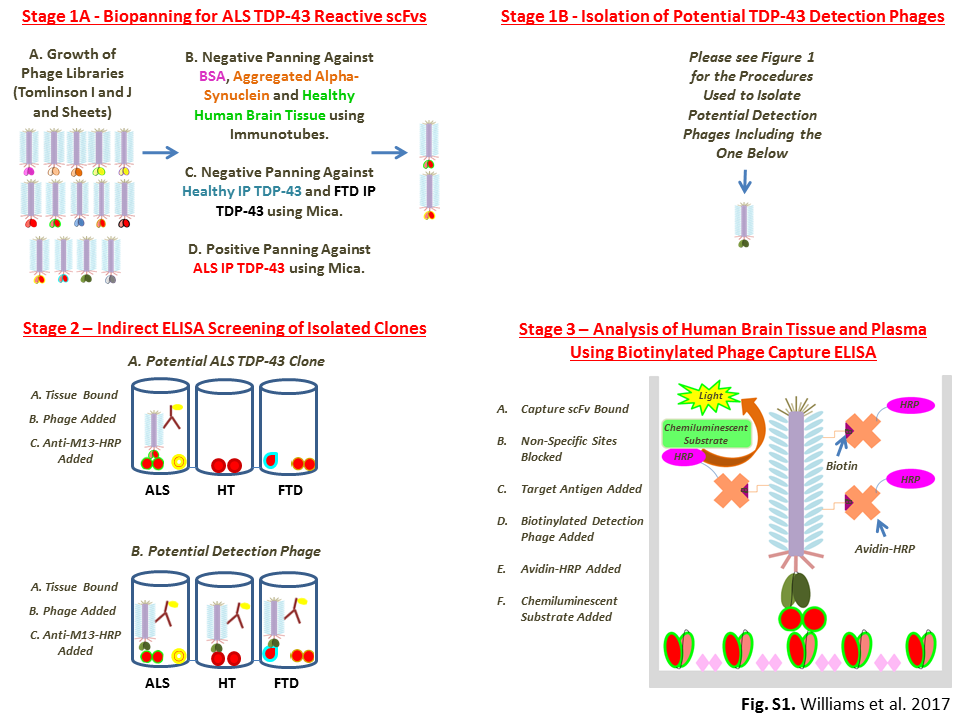

Supplement: Supplementary file 1 — Additional file 1: Figure S1. Schematic of the Development of our Biotinylated TDP-43 Phage Capture ELISA System. A schematic of the entire process utilized to develop our biotinylated TDP-43 phage-capture ELISA system for heightened detection of TDP-43 variants is shown. Stage 1A—Previously described AFM based negative and positive biopanning protocol to isolate the capture scFvs reactive with TDP-43 variants isolated from ALS brain tissue [32]. Starting with an initial scFv library, we first eliminated scFvs reactive with undesired targets including BSA, aggregated alpha-synuclein and healthy human brain tissue using immunotubes. AFM analysis was utilized to monitor the process. We next completed negative panning against TDP-43 immunoprecipitated from healthy and FTD brain tissue (motor cortex) using a mica surface to conserve limited sample availability. The remaining phages were then utilized in positive biopanning against TDP-43 immunoprecipitated from ALS brain tissue. Any eluted phages following this process should be specific for ALS related TDP-43 variants. Stage 1B—To isolate a detection phage that is reactive with all forms of TDP-43 we utilized the AFM biopanning process described in Fig. 1. Stage 2—Both the potential ALS TDP-43 reactive phages isolated in the previous study and the detection phages acquired in the current study were first screened in indirect ELISAs against ALS, FTD and healthy brain tissue. Any bound phage particles were detected with an anti-M13-HRP secondary antibody. ALS TDP-43 specific phages should generate signals only in the wells containing ALS tissue, whilst the detection phages should produce signals with all three tissue types. Stage 3 - Finally, following biotinylation of the 2700 coat protein on our detection phage to heighten our sensitivity, our complete phage capture ELISA system was used to analyze individual human brain tissue and plasma samples. Some of the utilized illustrations were adapted from previously publishe [file 12868_2017_334_MOESM1_ESM.tif]

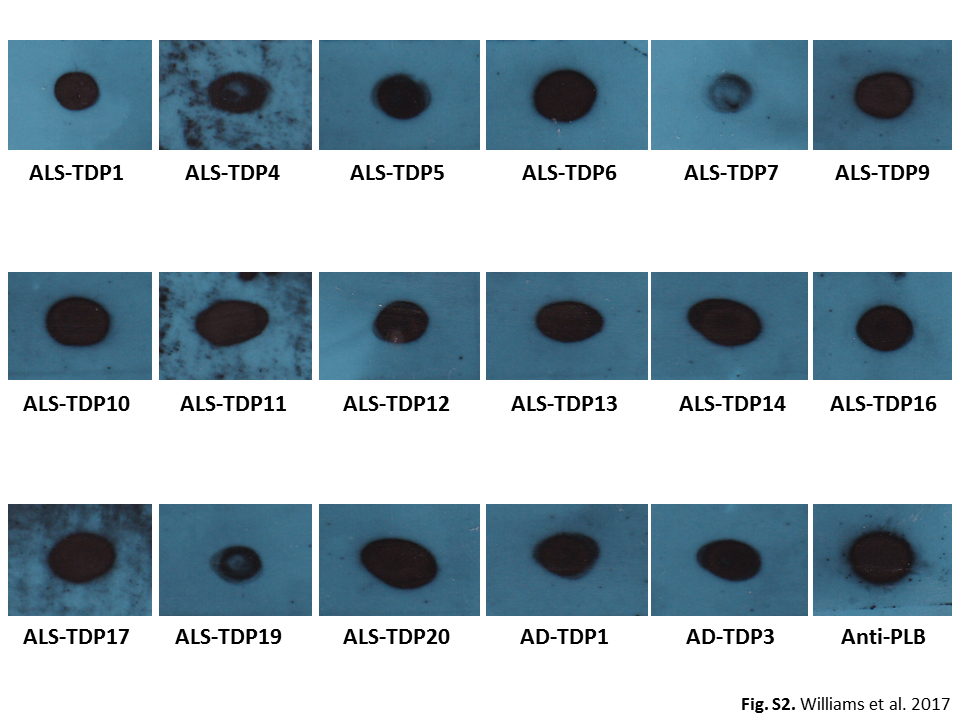

Supplement: Supplementary file 2 — Additional file 2: Figure S2. Expression of Soluble Antibody Fragments. Soluble antibody fragments from 17 of the 23 scFvs were produced and secreted into the supernatant as confirmed by dot blot analysis using an anti-c-myc antibody to label the scFvs. A scFv reactive with phosphorylase B was included as a positive control in the assay. [file 12868_2017_334_MOESM2_ESM.tif]

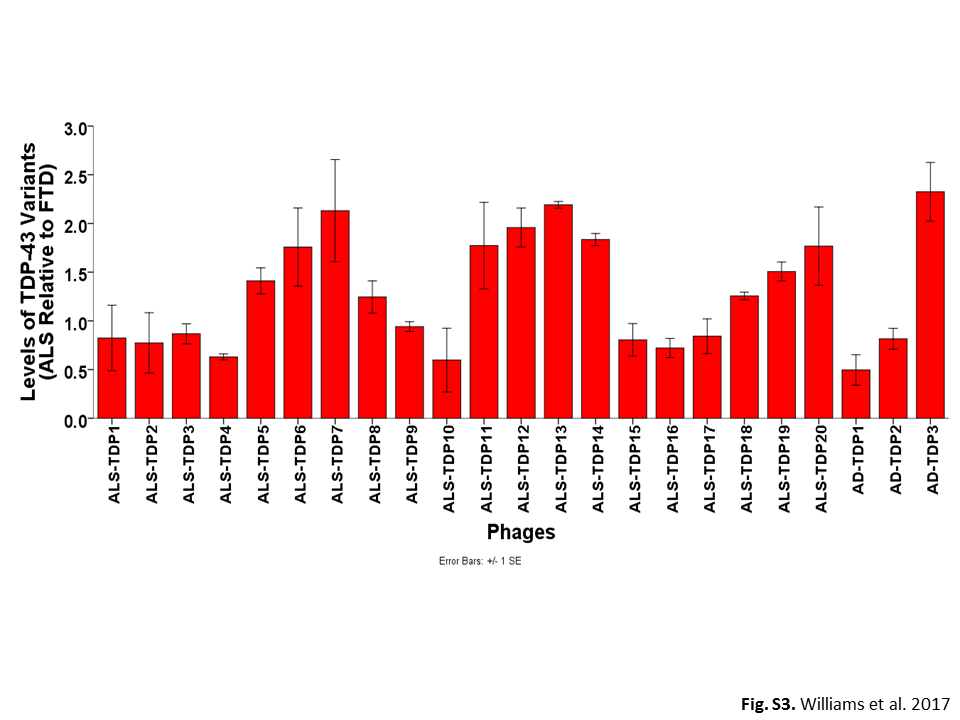

Supplement: Supplementary file 3 — Additional file 3: Figure S3. Signal Intensities of 23 Different Phages with Preferential Reactivity for ALS Related TDP-43 Variants. Indirect phage ELISAs using 23 different previously isolated clones with preferential reactivity for ALS TDP-43 variants were completed with homogenized brain tissue from ALS, FTD and healthy samples (motor cortex). All the phages generated increased reactivity with ALS brain tissue compared to both FTD and control cases. The ratio of ALS to control tissue for each phage was first calculated, followed by subtraction of the binding ratio produced with FTD brain tissue. The results of ALS relative to FTD are shown. Bound phages are detected with an anti-M13-HRP antibody. [file 12868_2017_334_MOESM3_ESM.tif]

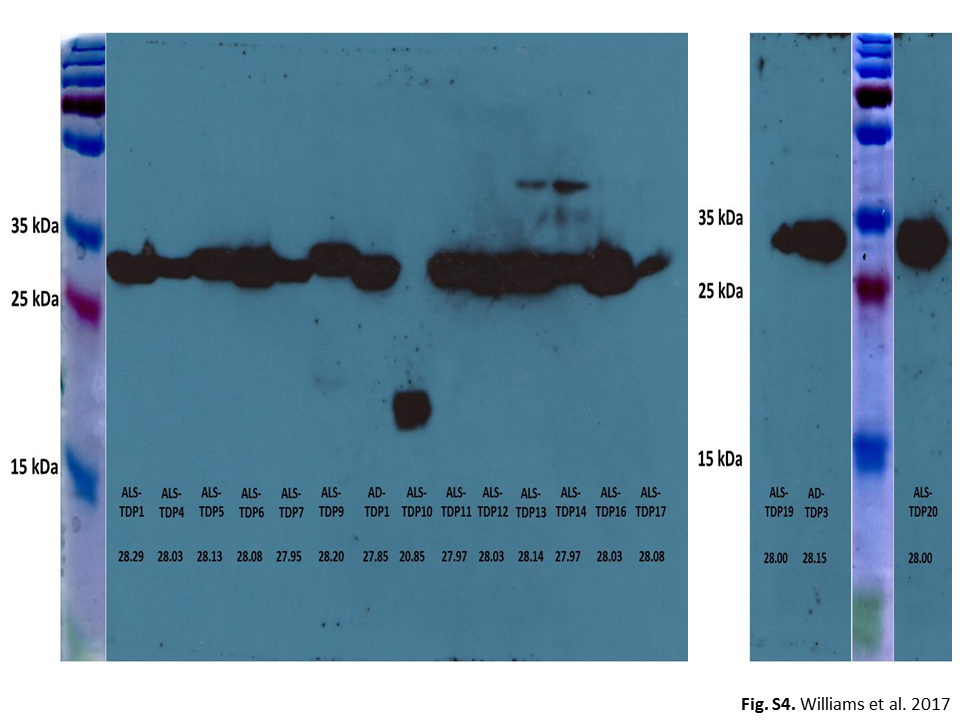

Supplement: Supplementary file 4 — Additional file 4: Figure S4. Analysis of Purified Soluble Antibody Fragments by Western Blot. Following Ni–NTA purification of 17 of the 23 scFvs, analysis by Western blotting indicated the presence of the expected ~28 kDa band corresponding to a full length scFv (except for ALS-TDP10). The estimated molecular weight for each scFv is indicated below their respective band. [file 12868_2017_334_MOESM4_ESM.tif]
