# Supplementary material for: TDP-43 protein variants as biomarkers in amyotrophic lateral sclerosis
Source: BMC Neurosci. 2017 Jan 25;18:20. doi: 10.1186/s12868-017-0334-7 (PMC5264476; doi:10.1186/s12868-017-0334-7)
Supplement: Supplementary file 5 — Additional file 5: Table S1. TDP-43 Protein Variants in Human Brain Tissue. [file 12868_2017_334_MOESM5_ESM.docx]

**Supplementary Table 1. TDP-43 Protein Variants in Human Brain Tissue**

| **Cases** | **ALS-TDP4** | **ALS-TDP5** | **ALS-TDP6** | **ALS-TDP9** | **ALS-TDP10** | **ALS-TDP11** | **ALS-TDP13** | **ALS-TDP14** | **ALS-TDP15** | **ALS-TDP20** | **AD-TDP1** | **AD-TDP 3** |
| --- | --- | --- | --- | --- | --- | --- | --- | --- | --- | --- | --- | --- |
|  |  |  |  |  |  |  |  |  |  |  |  |  |
| **ALS 1** | **+++++++** | **++++** | **+++++++** | **+++** | **++++++** | **+++++** | **++** | **++++++++++** |  |  | **++++** |  |
|  |  |  |  |  |  |  |  |  |  |  |  |  |
| **ALS 2** | **+++++++++** |  | **++++++++++++++++++++++++++** |  | **+++** | **++++** | **+++** | **++++++++** | **+++++++** |  | **++++++++** | **++++++** |
|  |  |  |  |  |  |  |  |  |  |  |  |  |
| **ALS 3** | **++++++** | **+++** | **+++++++++++++++++++++++++** | **+++++** | **+++** | **+++++** | **++** | **+++++** | **+++++++++** | **++** | **++++++** | **++++++++++** |
|  |  |  |  |  |  |  |  |  |  |  |  |  |
| **ALS 4** |  | **++** | **+++++++++++++** | **++++++++** | **++** |  |  | **++** | **+++++++** | **+++** |  | **++++** |
|  |  |  |  |  |  |  |  |  |  |  |  |  |
| **ALS 5** | **+++++** | **++** | **+++++** | **++** | **++** |  |  | **+++** | **++++** | **++** |  | **+++++** |
|  |  |  |  |  |  |  |  |  |  |  |  |  |
| **C-1** |  |  |  |  |  |  |  |  |  |  |  |  |
| **C-2** |  |  |  |  |  |  |  |  |  |  |  |  |
| **C-3** |  |  |  |  |  |  |  |  |  |  |  |  |
| **C-4** |  |  |  |  |  |  |  |  |  |  |  |  |
| **C-5** |  |  |  |  |  |  |  |  |  |  |  |  |

Note: The first “+” sign denotes being more than 1 SD above the controls with each additional “+” sign indicating a one SD increase. Only samples with 2 or more SDs are indicated.
