# Supplementary material for: TDP-43 protein variants as biomarkers in amyotrophic lateral sclerosis
Source: BMC Neurosci. 2017 Jan 25;18:20. doi: 10.1186/s12868-017-0334-7 (PMC5264476; doi:10.1186/s12868-017-0334-7)
Supplement: Supplementary file 6 — Additional file 6: Table S2. TDP-43 Protein Variants in Human Plasma. [file 12868_2017_334_MOESM6_ESM.docx]

**Supplementary Table 2. TDP-43 Protein Variants in Human Plasma**

| **Cases** | **ALS-TDP3** | **ALS-TDP5** | **ALS-TDP6** | **ALS-TDP7** | **ALS-TDP10** | **ALS-TDP11** | **ALS-TDP15** | **ALS-TDP17** | **AD-TDP2** |
| --- | --- | --- | --- | --- | --- | --- | --- | --- | --- |
|  |  |  |  |  |  |  |  |  |  |
| **SALS-1** | **++** | **++++** | **++++++** | **++++++** | **+++** | **+++** | **+** | **++++** | **+++++** |
| **SALS-2** | **++** | **++** | **+++++** | **+++++** | **++** | **++** | **+** | **++** | **++** |
| **SALS-3** | **+** | **+** | **+++++** | **++++** | **+** | **+** | **+** | **+++** | **+** |
| **SALS-4** | **++** | **++++** | **++++++++++** | **++++++++** | **+++** | **++++** | **+** | **++++** | **+++++** |
|  |  |  |  |  |  |  |  |  |  |
| **c9orf72-1** |  | **++** | **++** | **++** | **+++** | **+** | **+** | **+** |  |
| **c9orf72-2** |  |  |  |  |  |  |  |  |  |
| **c9orf72-3** |  |  |  | **+** | **+** |  |  |  |  |
| **c9orf72-4** |  | **++** | **++++** | **++++++** | **++** | **+++** |  | **+** | **++** |
|  |  |  |  |  |  |  |  |  |  |
| **C-1** |  |  |  |  |  |  |  |  |  |
| **C-2** |  |  |  |  |  |  |  |  |  |
| **C-3** |  |  |  |  |  |  |  |  |  |

Note: The first “+” sign denotes being more than 1.5 SD above the controls with each additional “+” sign indicating a one SD increase. Only samples with 1.5 or more SDs are indicated.
